# Severe unilateral orbitopathy in a patient with Hashimoto’s thyroiditis - a case report

**DOI:** 10.1186/s12886-018-1018-5

**Published:** 2019-01-08

**Authors:** Ewa Cyranska-Chyrek, Michal Olejarz, Ewelina Szczepanek-Parulska, Piotr Stajgis, Anna Pioch, Marek Ruchala

**Affiliations:** 1 Department of Endocrinology, Metabolism and Internal Medicine, Poznan University of Medical Sciences, Przybyszewskiego 49, 60-355 Poznan, Poland; 2 Department of General Radiology and Neuroradiology, Poznan University of Medical Sciences, Przybyszewskiego 49, 60-355 Poznan, Poland

**Keywords:** Orbitopathy, Ophthalmopathy, Hashimoto’s thyroiditis, Magnetic resonance

## Abstract

**Background:**

Thyroid-associated orbitopathy (TAO) constitutes an immune-mediated inflammation of the orbital tissues of unclear etiopathogenesis. TAO is most prevalent in hyperthyroid patients with Graves’ disease (GD); however, severe cases of orbitopathy associated with Hashimoto’s thyroiditis (HT) have rarely been described.

**Case presentation:**

Herewith we report an unusual case of a middle-aged clinically and biochemically euthyroid woman with a stable HT, who developed a severe unilateral left-sided TAO. Thyrotropin receptor antibodies (TRAb) concentration was negative. Intraocular pressure in the left eye was mildly elevated (24 mmHg), while vision acuity was not compromised. Abnormal positioning of the eyeball suggested the extraocular muscles involvement. Unilaterally, von Graefe’s, Stellwag’s, Kocher’s and Moebius' signs were positive. Conjunctival erythema, redness and edema of the eyelid and an enlarged, swollen lacrimal caruncle were visible. She received 4/7 points in the Clinical Activity Scale (CAS) and class IV in the NO SPECS severity scale for the left eye (I-0, II-a, III-0, IV-b, V-0, VI-0). Magnetic resonance imaging (MRI) revealed thickening of the left medial rectus muscle with an increase in T2 signal intensity and prolonged T2 relaxation indicating an active form of TAO. The patient received therapy with glucocorticosteroids intravenously, followed by intramuscular injections with a cumulative dose of 3.24 g of methylprednisolone during a 9-week period with good tolerance. The applied therapy, combined with adequate L-thyroxine substitution, as well as vitamin D and selenium supplementation, resulted in a complete remission of ophthalmic symptoms.

**Conclusions:**

Unilateral exophthalmos in TRAb-negative patients with HT is not a typical manifestation of the disease, and requires a wider differential diagnosis with MRI of the orbits. Scheme of three iv. pulses of methylprednisolone intravenously and the continuation of treatment with im. injections seems to be an effective and safe method of treatment in this group of patients. What is more, adequate vitamin D supplementation and the maintenance of biochemical euthyroidism may help to achieve an ultimate therapeutic effect. Patients with TAO in the course of HT need a careful and continued interdisciplinary approach both ophthalmological and endocrinological. Further studies are needed to elucidate the etiopathogenesis of TAO in TRAb-negative patients.

## Background

Thyroid-associated orbitopathy (TAO) constitutes an immune-mediated inflammation of the orbital tissues of unclear etiopathogenesis [1]. The process involves extraocular muscles, surrounding orbital connective tissue and retro-orbital fat. These lesions may lead to exophthalmos, soft periocular tissue edema, and eyeballs mobility disturbances. Symptoms reported by patients may include pain in the eyes, irritation, excessive tearing, diplopia and reduced vision acuity [2], exerting strong negative impact on the quality of life [3]. Furthermore, severe orbitopathy may cause optic nerve compression leading to sight disturbances, or even loss of sight. The disease usually affects both eyes, while only about 10–14% of patients present with unilateral orbitopathy. Nevertheless, the severity of TAO is not determined by its symmetric or asymmetric presentation. Thus, one-sided TAO should not be regarded as a milder form of the disease [4].

An annual incidence rate of TAO for women is estimated at 16 cases per 100,000 population, and 2.9 cases per 100,000 population for men [5]. Milder forms occur even in up to 50% of patients with GD, whereas severe forms affect 3 to 5% of this population [6]. Even though TAO is most prevalent in hyperthyroid patients with GD, it might also occur in euthyroid, or hypothyroid patients with Hashimoto thyroiditis (HT), or even in subjects without any clinically manifested thyroid diseases [7]. Moreover, according to a recent study by Ponto et al., euthyroid and hypothyroid patients account for 4.3% of all TAO cases [8]. Clinically overt TAO occurs in about 6% of patients with HT; nevertheless, severe cases have been very rarely described in the literature [8]. In this specific subgroup of patients, TAO is usually milder, has a less pronounced influence on the quality of life, and is hardly ever accompanied by serious complications, or constitutes a threat to the sight; hence, it can be easily mistaken for another orbital pathology.

Herewith we report an unusual case of a middle-aged clinically and biochemically euthyroid woman with a stable HT who developed a severe unilateral TAO.

## Case presentation

A 41-year-old female was admitted to hospital because of unilateral proptosis in the left eye developing for about six-months. She had suffered from HT for the past 2 years and had been treated with levothyroxine 25 μg daily. She did not present any other significant comorbidities and had never smoked. Her previous personal and family history was negative for thyroid disorders. Laboratory results indicated euthyroidism - TSH level was 2.67 μU/ml (reference range 0.27–4.20 μU/ml), the free T3 and free T4 concentrations were 4.97 pmol/l (reference range 3.90–6.70 pmol/l) and 13.58 pmol/l (reference range 11.5–21.0 pmol/l), respectively. Thyrotropin receptor antibodies (TRAb) concentration was normal (TRAb 0.9 IU/l, reference range < 2). However, anti-thyroid peroxidase (TPOAb) serum levels and anti-thyroglobulin autoantibodies (TgAb) were significantly elevated: 279 IU/ml (reference range 0–34 IU/ml) and 194 IU/ml (reference range 10–115 IU/ml), respectively. The patient’s 25-OH vitamin D serum level was 25 ng/ml indicating mild vitamin D deficiency. Ultrasound examination demonstrated a thyroid gland with features suggesting chronic autoimmune thyroiditis (heterogeneous decreased echogenicity, no focal lesions, normal size and vascularity).

**Fig. 1:**
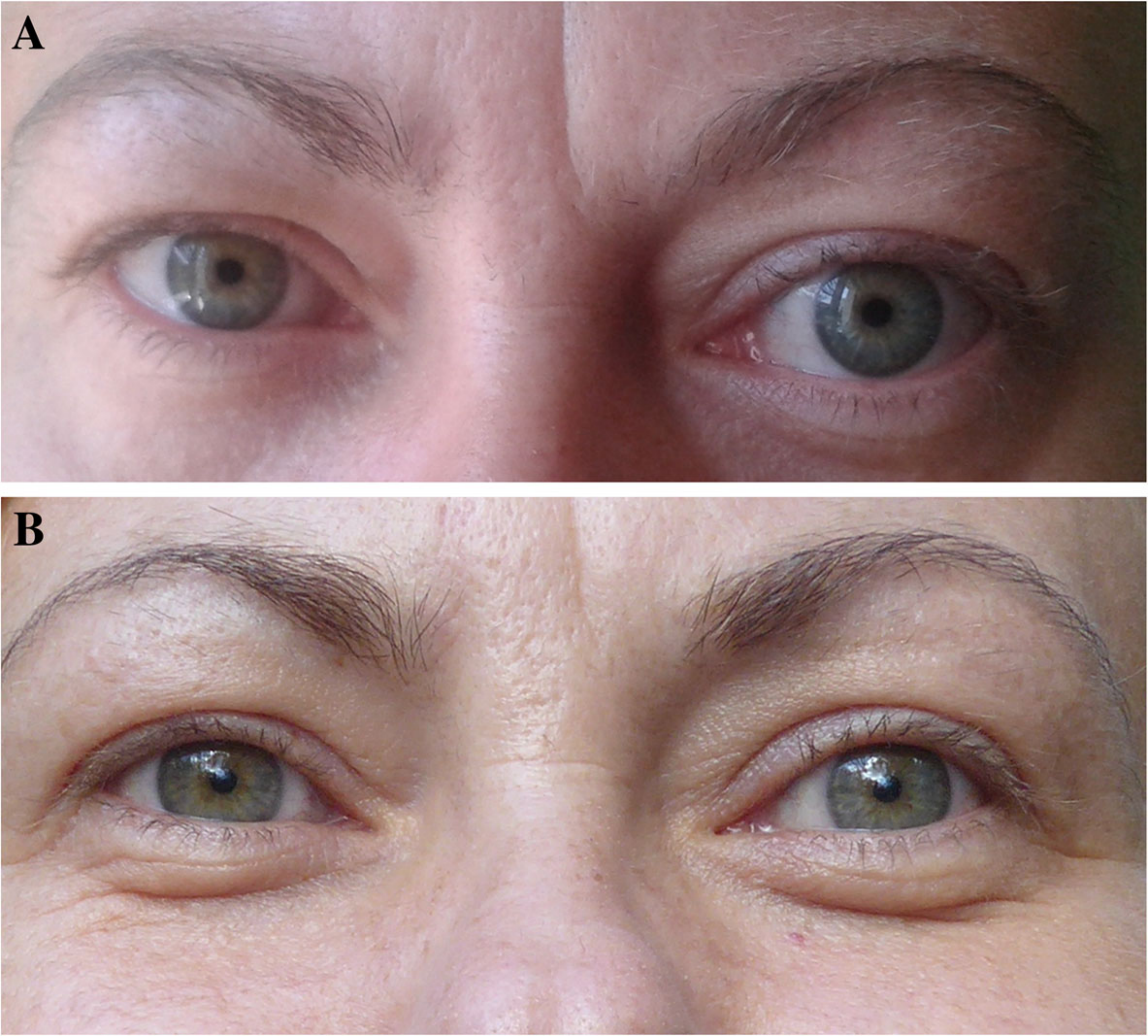
**a**. Patient's eyes on first admission to the hospital. **b**. External view of the patient 6 months following glucocorticosteroid therapy

In the neutral position, the left eyeball was positioned convergently and downwards which implied extraocular muscle involvement. In addition, the patient also presented conjunctival erythema, eyelid redness and edema, and an enlarged, swollen lacrimal caruncle (Fig. 1). Von Graefe’s, Stellwag’s, Kocher’s and Moebius' signs were positive in the left eye, whereas the Rosenbach’s sign was negative. On ophthalmic examination, vision acuity was not compromised (6/6 in both eyes). The intraocular pressure was mildly elevated in the left eye (24 mmHg), and it was normal in the right eye (19 mmHg). The patient received 4/7 points in the Clinical Activity Scale (CAS) due to the eyelid edema, redness, conjunctival injection, and inflammation of left caruncle. Furthermore, the severity of TAO was classified as IV in the NO SPECS scale due to extraocular muscles involvement in the left eye (I-0, II-a, III-0, IV-b, V-0, VI-0). In terms of the right eye, she received 0/7 points in CAS, and class 0 in NO SPECS scale.

**Fig. 2:**
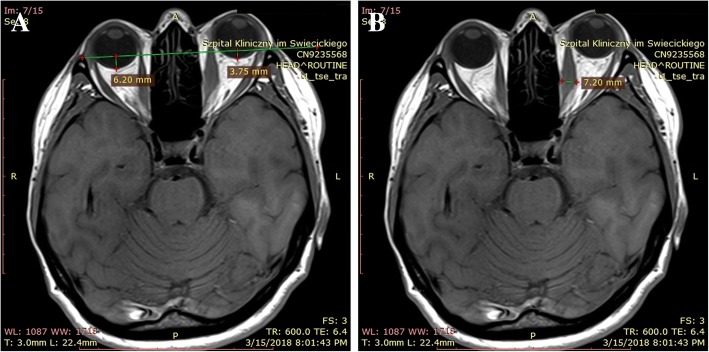
**a**. Left-sided exophthalmos demonstrated by MRI in an axial plane. **b**. Thickening of the left medial rectus muscle demonstrated by MRI in a transverse plane

**Fig. 3:**
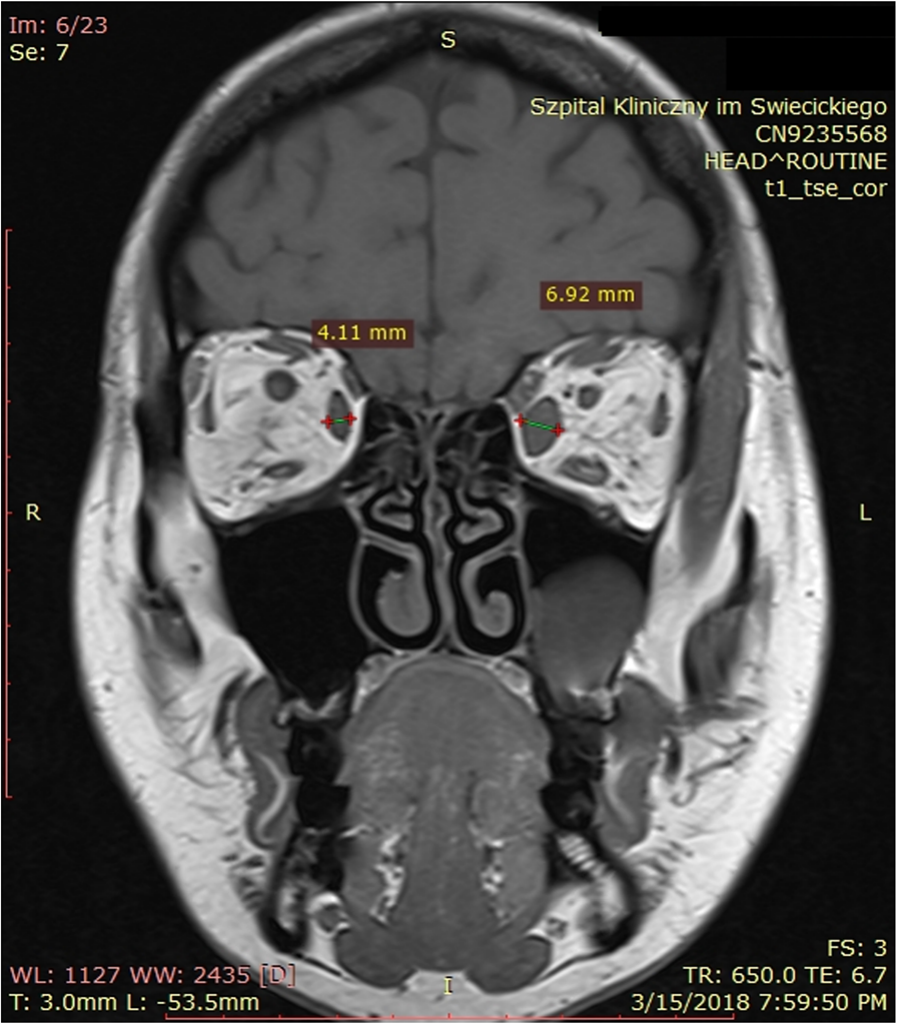
Thickening of the left medial rectus muscle demonstrated by MRI in a coronal plane. In addition, slight thickening of the inferior rectus muscle is also visible. Besides, a large cyst in the left maxillary sinus is visible sized 23 mm

**Fig. 4:**
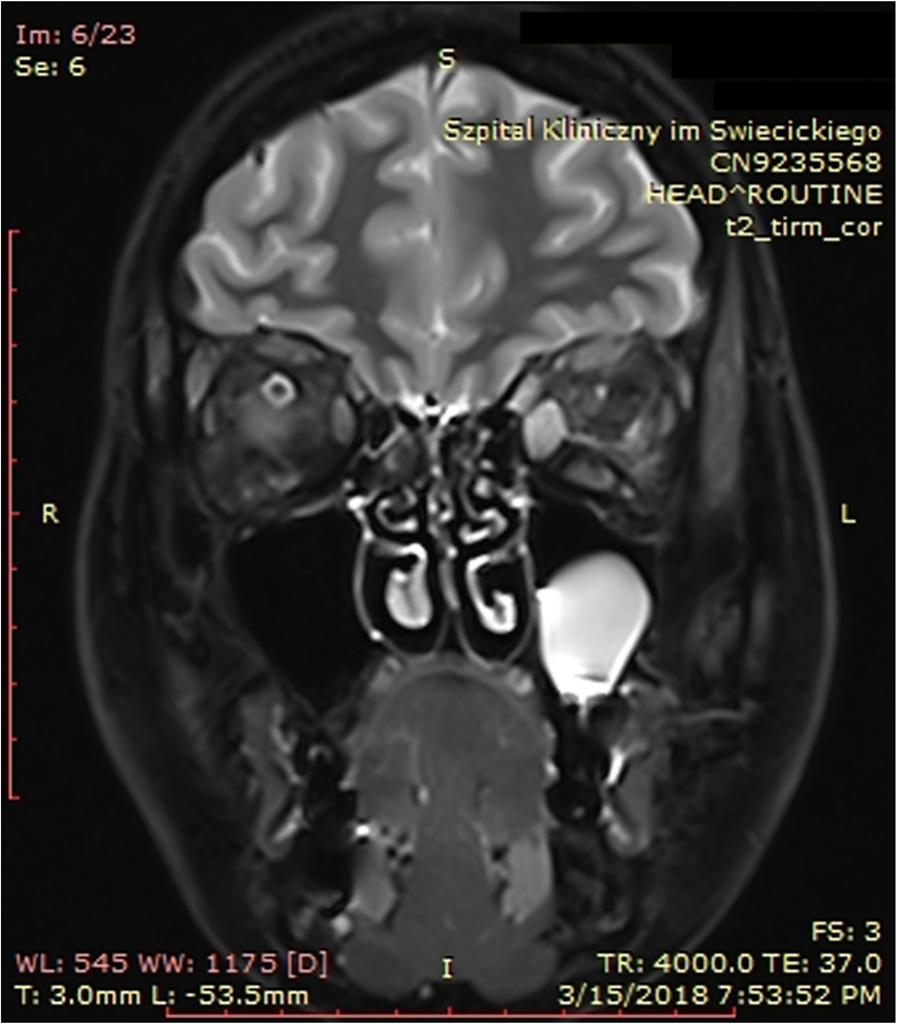
Increased T2 signal intensity from the left medial rectus muscle on MRI in a coronal plane

**Fig. 5:**
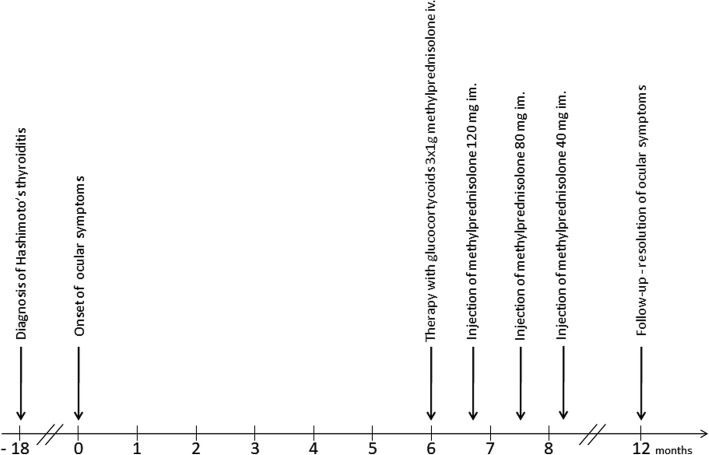
The timeline representing all important steps in patient’s diagnosis and therapy

Magnetic resonance imaging (MRI) of the orbits revealed unilateral left-sided exophthalmos (Fig. 2), predominantly caused by the thickening of the medial rectus muscle (Fig. 3), with an increase in T2 signal intensity (Fig. 4) and prolonged T2 relaxation, indicating an active form of TAO. On MRI a 23 mm cyst was found inside the left maxillary sinus. CRP concentration was 0.3 mg/l (normal < 5 mg/l), the patient had no history of sinusitis, and presented no clinical symptoms on laryngological examination. The therapy included the administration of glucocorticosteroids intravenously according to the scheme designed and widely applied at our department, i.e. three pulses of intravenous methylprednisolone (1 g of methylprednisolone per pulse on three consecutive days), followed by an intramuscular injection regimen: one dose every three weeks (120 mg, 80 mg, 40 mg), i.e. a cumulative dose of 3.240 g of methylprednisolone during a 9-week follow-up period. The therapy was very well tolerated, with no severe side-effects observed. Simultaneously, the levothyroxine dose was increased from 25 to 50 μg per day in order to keep TSH in the lower half of the normal range. Additionally, the patient also received selenium supplementation (200 μg daily) and vitamin D (4000 IU daily). The administered therapy resulted in a complete remission of ophthalmic symptoms, and the patient has been free of symptoms in the course of a 6-month follow-up period (Fig. 1). The timeline representing all vital steps in the patient’s diagnosis and therapy is presented in Fig. 5.

## Discussion and conclusions

To date only a few studies regarding the issue of TAO accompanying HT have been conducted. The first study on the prevalence of ophthalmic symptoms in patients with HT was published by Tijang et al. who evaluated a group of 20 patients with HT, and found that eye signs were prevalent in about 34% of patients [9]. In another study, on 110 patients with HT, Kan et al. found eye symptoms to be present in about 22.7% of individuals with HT. It is of note that the symptoms were predominantly mild, and out of the studied cohort of patients, only two subjects achieved a CAS score of ≥4 out of 5 symptoms proposed by Mourits et al. [10, 11]. Thus far, the latest and largest study regarding this matter by Kahaly et al. was performed on 700 patients with HT, and demonstrated that clinically overt TAO was present in 6% of patients [12]. The same study also indicated that patients with HT and coexisting TAO compared to patients without eye symptoms were older, had a longer duration of HT, were heavier smokers, and were less likely to suffer from another autoimmune disease [12]. However, the abovementioned facts are not confirmed in our patient, since she is a middle-aged non-smoker without accompanying autoimmune disorders.

TAO occurs most commonly in GD where its pathophysiology has already been extensively studied and is said to be related to the presence of TRAb. The thyrotropin receptor is expressed not only in the thyroid follicular cells, but also in the orbital fibroblasts [13, 14]. The autoimmunity directed against the receptor on orbital tissues induces infiltration of inflammatory cells, hyaluronic acid accumulation, and the orbital adipose tissue expansion which eventually leads to the orbital connective tissues remodeling and fibrosis in its final stage [15, 16]. The thyrotropin receptor theory can also explain the occurrence of TAO in patients with HT and positive testing for Thyroid Stimulating Antibodies (TSAb). As pointed out in the paper by Kahaly et al., 68% of patients with clinically overt TAO and HT had elevated TSAb, as compared to only 5.5% of patients with HT and no signs of TAO. Moreover, the study also demonstrated that TSAb levels were significantly higher in patients with severe TAO in comparison with those with mild TAO [12]. However, the pathogenesis of TAO in patients who are negative for TRAb/TSAb testing remains still unclear. According to some data, one of the possible explanations of this phenomenon could be autoimmunity against skeletal muscle calsequestrin in extraocular and eyelid muscles, as well as against collagen XII in orbital fibroblast cell membranes [17]. In addition, several studies have demonstrated low vitamin D status in patients with HT. In fact, in numerous research, low vitamin D levels were associated with the presence of antithyroid autoantibodies and abnormal thyroid function tests [18–20]. However, to date very few studies have been conducted on the potential role of vitamin D deficiency in the development of TAO. A recent prospective randomized study by Elewa et al. indicated that low baseline vitamin D concentration was related to exophthalmos in hyperthyroid patients with GD. In this trial, vitamin D supplementation conducted together with methimazole therapy was superior to methimazole alone in reducing the severity of exophthalmos [21]. In a retrospective study, Lahooti et al. found that vitamin D deficiency represents a risk factor for TAO in GD, but not in HT. This might suggest a different pathogenesis of TAO in those two entities [22]. However, these findings require confirmation in larger prospective trials.

Even though unilateral exophthalmos tends to occur more often in euthyroid and hypothyroid patients, it is still not a typical manifestation of TAO and requires a wider differential diagnosis [8]. In such groups of patients, orbital neoplasms, such as lymphoma, benign tumors (e. g. rhabdomyomas, hamartomas) and metastases to the orbit, should be primarily excluded. Furthermore, specific orbital inflammation (e. g. due to sarcoidosis or systemic lupus erythematosus), orbital myositis, vascular malformations, infections, neuromuscular dysfunctions, orbital pseudotumor/non-specific orbital inflammation, cavernous sinus thrombosis, sphenoid meningioma and IgG4-related ophthalmic disease also require consideration [23–25]. Unfortunately, the patient’s IgG4 concentration level was not measured which constitutes a limitation of our report. However, our patient had a negative history of any rheumatoid disorders, no signs of any other organs involvement, while the MRI picture was not typical of IgG4 disease presentation (i.e. lacrimal glands were not involved). In fact, MRI is a method of choice in a differential diagnosis of TAO, due to the great potential of tissue differentiation, and the capability of multiple plane imaging with no exposure to ionizing radiation [26].

Minor eye symptoms associated with thyroid disease seem to occur quite often. The most common eye sign in patients with HT is the upper eyelid retraction, while the most common symptom is retrobulbar pain [9, 10]. However, severe TAO in the course of HT remains a rare finding. In fact, most cases published to date more commonly describe patients with bilateral than unilateral eye involvement, predominantly with positive testing for TRAb, and a very good response to systemic intravenous steroid therapy [27–29]. Yoshihara et al. reported two cases of patients with severe TAO and negative TRAb results who were treated in a different way - with oral glucocorticoid therapy and orbital irradiation at the dose of 15 Gy. The treatment resulted in a gradual improvement [30].

Therapy with intravenous glucocorticoids comprises the first line therapy for moderate to severe TAO [31]. It has a response rate of approximately 70–80% (compared to 50–60% for oral therapy) and also causes significantly fewer adverse events than glucocorticoids administered via oral route [1, 32]. The European Thyroid Association/European Group on Graves’ Orbitopathy 2016 Guidelines recommends starting doses of 0.5–0.75 g methylprednisolone weekly for 6 weeks, followed by 0.25–0.5 g methylprednisolone for another 6 weeks in the treatment of severe cases of Graves’ orbitopathy. The cumulative dose of 8 g in 12 weeks should not be exceeded [1, 32]. As yet, dedicated guidelines for treating orbitopathy in HT have not been published. The starting glucocorticosteroids doses used for the treatment in so far published cases of severe TAO in HT varied from 1 up to 5 g of methylprednisolone in the first week of treatment, and were followed by significantly tapered doses over several weeks [27–29]. In our patient, three doses of 1 g methylprednisolone pulse therapy on three consecutive days were administered. The dosage was in the mid-range of the previously described doses in similar patients [27–29]. What is more, in studies conducted on patients with GD, apart from glucocorticoid treatment, selenium seemed to have a beneficial effect on the course of a moderate or mild orbitopathy. Additionally in HT, selenium appears to decrease local inflammatory reactions, lower the TPOAb production, as well as improve thyroid morphology [33]. Thus, adding selenium to the therapy of TAO in HT might have a potentially beneficial effect on ophthalmic symptoms in the course of HT.

Even though minor ocular symptoms are quite prevalent in patients with HT, severe unilateral orbitopathy, particularly in the absence of TRAb/TSAb, occurs extremely rarely. It is important to bear in mind this uncommon entity, since proper treatment usually results in the positive outcome and prevents from serious, potentially sight-threatening complications.

In conclusion, our report presents a rare case of unilateral severe TAO in the course of HT in the absence of TRAb. Unilateral exophthalmos in patients with HT who are TRAb-negative is not a typical manifestation of the disease and requires a wider differential diagnosis with MRI of the orbits. The scheme of three pulses of methylprednisolone intravenously and the continuation of treatment with intramuscular injections seems to be an effective and safe method of therapy in such patients. Furthermore, adequate vitamin D supplementation and the maintenance of biochemical euthyroidism may help to achieve an ultimate therapeutic effect. Patients with TAO in the course of HT need a careful and continued interdisciplinary approach both ophthalmological and endocrinological. Further studies are needed to elucidate the etiopathogenesis of TAO in TRAb-negative patients.

## Abbreviations

CAS: Clinical Activity Scale
GD: Graves’ disease
HT: Hashimoto’s thyroiditis
im.: Intramuscularly
iv.: Intravenously
MRI: Magnetic resonance imaging
TAO: Thyroid-associated orbitopathy
TRAb: Thyrotropin receptor antibodies
TSAb: Thyroid Stimulating Antibodies
TSH: Thyrotropin
